# Antimicrobial and toxicological evaluation of the leaves of Baissea axillaries Hua used in the management of HIV/AIDS patients

**DOI:** 10.1186/1472-6882-6-22

**Published:** 2006-06-21

**Authors:** Tavs A Abere, Freddy O Agoreyo

**Affiliations:** 1 Department of Pharmacognosy, Faculty of Pharmacy, University of Benin, Benin-City, Nigeria; 2 Department of Anatomy, College of Medical Sciences, University of Benin, Benin City, Nigeria

## Abstract

**Background:**

Persistent diarrhea is a common endemic disease with high incidence among the Africans including Nigerians. It also represents a frequent opportunistic disease in people living with HIV. Diarrhea represents one of the most distressful and persistent symptoms of HIV/AIDS, which may or may not be accompanied by an infection. The leaves decoction of *Baissea axillaries *Hua (Apocynaceae) is used by traditional herbalists in Edo state, Nigeria for the management of people living with HIV/AIDS. Determination of its antimicrobial activity and toxicological profile will provide supportive scientific evidence in favour of its continuous usage.

**Method:**

Chemical and chromatographic tests were employed in phytochemical investigations. Inhibitory activities of aqueous and ethanolic extracts against clinical strains of *Escherichia coli*, *Pseudomonas aeruginosa*, *Staphylococcus aureus *and *Streptococcus faecalis *were compared with Togamycin (Spectinomycin). Our report includes minimum inhibitory concentration (MIC) against the test organisms. Toxicological evaluation was determined by administering 250 mg/kg and 500 mg/kg of extracts on male Wister rats for 14 days with normal saline as control. The kidneys, liver, heart and testis tissues were examined.

**Results:**

Phytochemical studies revealed the presence of alkaloids, tannins, and cyanogenetic glycosides. The extracts inhibited the growth of *Escherichia coli*, *Pseudomonas aeruginosa *and *Staphylococcus aureus *to varying extents, but only the ethanolic extract inhibited growth in *Streptococcus faecalis*. The LD_50 _of the extract in mice was above 5000 mg/kg body weight when administered intraperitoneally. Toxicological evaluation showed mere ballooning degeneration of the liver at 250 mg/kg while at 500 mg/kg there was tissue necrosis. The low and high doses showed ill-defined leydig cells in the testis and no remarkable changes in the heart and kidneys.

**Conclusion:**

Extracts of *Baissea axillaries *have demonstrated antimicrobial activity against clinical strains of selected microorganisms. While there is toxicity at the dose of 500 mg/kg, the therapy shows potential for application in the treatment of diarrhoea associated with AIDS/HIV. Further studies of *Baissea axillaries *on diarrhoea and toxicity are necessary to evaluate its mechanism of action and to fully establish its safety profile.

## Background

In Nigeria, where traditional medicine, with its cheap resources, is the main source of health-care, the traditional medical practitioners (TMP) are the main medical manpower available and accessible to the rural dwellers. Hence HIV/AIDS patients in such rural settings would be expected to consult the traditional healers privately as the first appeal for management of various opportunistic infections at a very affordable cost. A greater driving force to this decision might arise from the fact that no known western drugs can produce cure and therefore, People living with HIV/AIDS (PLWHA) are faced with the belief that a possible cure might be found in traditional medicine [1].

Persistent diarrhea (or dysentery) is a common endemic disease with high incidence among Africans including Nigerians. Symptoms of dysentery (an infection by amoeboid microorganism) consist of a strong urge for little stools, which occur frequently and usually accompanied by mucous and/or blood. It also represents a frequent opportunistic disease in people living with HIV. Diarrhea is more common than dysentery and is a major opportunistic disease with high incidence among the people living with HIV, with a long duration of over one month, often found in those infected with HIV-1 strain of the virus. Diarrhea represents one of the most distressful and persistent symptoms of HIV/AIDS, which may or may not be accompanied by infection. It is characterized by tonic spasm of the gastrointestinal tract, frequent watery stools and incessant loss of body fluid leading to dehydration.

Apocynaceae, is a family of 315 genera and 2,000 species and is closely allied to Ascledpiadeies. They are collectively known as the milkweed family [2]. *Baissea axillaries *known as imu (Yoruba); otunta (Ibo) and ewuonkwonegie (Benin) is a familiar creeping climber found on fences and trees in Nigeria and grows to a very long length. It is popular with herbalists who mixed it with other ingredients and administer as a concoction for people living with HIV/AIDS. *Baissea axillaries *is also used for infertility in women, as an anti-ulcer and an anti-hypertensive. In Philippines Island, it is used as tea by diabetic natives, and apparently served as a successful substitute for the unavailable insulin [3].

A strong appeal of alternative medicine no doubt is the perception that its use of natural products and its methods of treatment are gentler and less hazardous than those employed by conventional medicine. However, a few members have been found nourishing while others were very distasteful or harmful due to toxic nature of the constituents of the plants [4]. Efforts are continually being taken by modern researchers to examine the merits of traditional medicine in the light of modern science with a view aimed at adopting effectively beneficial medical practice and discouraging harmful ones[5], hence there is need for information on the toxicity of these plants [6].

## Methods

### Plant collection and authentication

The leaves of *Baissea axillaries *Hua were collected in Ugbowo area of Benin City, Edo State, Nigeria on the 5^th ^of August, 2004. The plants were identified by the plant curator in the Department of Pharmacognosy herbarium, University of Benin, Benin City, where voucher specimens were deposited. The leaves were air-dried and powdered on an electric mill.

### Phytochemical studies

Chemical and Chromatographic tests were employed in the preliminary phytochemical screening for various secondary metabolites such as Tannins (phenazone; iron complex; formaldehyde and modified iron complex tests), Cardiac glycosides (keller killiani and kedde tests), Alkaloids (mayer's; dragendorff's; wagner's and 1% picric acid reagents), Saponin glycosides (frothing and haemolysis tests), Anthracene derivatives (borntrager's test for combined and free anthraquinones) and Cyanogenetic glycosides (sodium picrate paper test) [7-10]. Thin layer chromatography of aqueous and ethanolic extracts on Silica gel-G, activated by heating at 110°C for 30 minutes was developed with the solvent system Acetone: water: ammonia (90:7:3), viewed under UV light and sprayed with Dragendorff's spray reagent and R_f _values calculated. Paper chromatography of the two extracts, using the ascending method and Whatman No.3 mm was developed with the solvent system n-butanol: water: acetic acid (4:1:5), examined both in daylight and under UV light at 25 nm, sprayed with ferric chloride spray until colours developed and R_f _values calculated.

### Organisms

The organisms used were clinical isolates of *Escherichia coli*, *Pseudomonas aeruginosa*, *Staphylococcus aureus *and *Streptococcus faecalis *obtained from HIV positive patients in the University of Benin Teaching Hospital, Benin City.

### Preliminary screening

Powdered leaf material (2 kg) of *Baissea axillaries *Hua was divided into 1 kg each and extracted with water and ethanol respectively at room temperature. After 48 h, the extracts were clarified by filtration and evaporated to dryness *in vacuo*. Residues were collected and evaluated against the four test organisms at 80 mg/kg in 50% aqueous methanol. The results are shown in Table 1.

**Table 1:** Antimicrobial screening of *Baissea axillaries *leaves at 80 mg/mL, in methanol: water (1:1).

**Test organism**	Aqueous extract of *Baissea axillaries*	Ethanolic extract of *Baissea axillaries*	Togamycin (10 ug/ml)	Solvent Aq.methanol
	(mm)	(mm)	(mm)	
*Escherichia coli*	23	22	25	0
*Pseudomonas aeruginosa*	25	18	20	0
*Staphylococcus aureus*	9	9	24	0
*Streptococcus faecalis*	++	16	22	0

The diameter recorded is the diameter of the zone of inhibition less the diameter of the cup (8 mm).

### Antimicrobial test

The hole-in-plate agar diffusion method was used for the anti-microbial screening against the four test organisms, following standard procedures with nutrient agar as medium.

The minimum inhibitory concentration (MIC), the lowest concentration of a compound that inhibits growth of a microorganism, was determined by the standard two-fold dilution technique using nutrient broth medium [11].

### Acute toxicity test

The LD_50 _of the extracts was determined using Lorke's method [12] by administering extracts in normal saline to 20 mice using the intraperitoneal route.

### Evaluation of the toxicity

Thirty (30) wistar male rats were randomly distributed into three (3) groups of ten (10) rats each. The first group (A) served as control and received 0.9 ml of normal saline (solvent system). The second (B) and third (C) groups received 250 mg/kg and 500 mg/kg body weight of *Baissea axillaries *extract respectively. All rats in this evaluation received their respective doses daily and had free access to food and water at all times. They were observed for signs of toxicity (Pharmacological reactions, abnormal behaviours and general body conditions). At the end of 14 days, the rats were sacrificed by chloroform anaesthesia. The liver, kidney, hearts and testis were removed and preserved in 10% normal saline. Tissues from the organs were sectioned (6 microns thick) in paraffin wax and stained with hematoxylin and eosin (H & E) for assessment of tissue morphology. Results are presented in table 4.

## Discussion

Many plant products are being used by HIV/AIDS patients and traditional practitioners in Edo State, and elsewhere in Nigeria, without any scientific proof that they possess anti-HIV activity. However we have been making efforts to convince traditional healers to offer their remedies for scientific evaluation and this has certainly foster good collaboration between us in the quest for a treatment for HIV/AIDS from natural products. The result of this is an array of medicinal plants which serves to reduce the incidence of opportunistic infections and as potential sources of anti-HIV agents.

Phytochemical screening revealed the presence of alkaloids, tannins and cyanogenetic glycosides. Both aqueous and ethanolic extracts were submitted to antimicrobial tests at 80 mg/ml so as to mimic the administration of *Baissea axillaries *by the traditional healers and to also locate the fraction with the higher activity. The results indicated that both extracts demonstrated potent antibacterial activity against *Escherichia coli *and *Pseudomonas aeruginosa *compared with Togamycin, a brand of spectinomycin frequently used in the University of Benin Teaching, Benin City. Among other possible causative microorganisms implicated in diarrhea, the role of enteroaggregative *Escherichia coli *has been reported. The results also indicated that while both extracts exhibited mild antibacterial activity against *Staphylococcus aureus*, only the ethanolic extract was active against *Streptococcus faecalis*. MIC values of *Baissea axillaries *leaves for the test organisms are given in Table 2.

**Table 2:** Minimum inhibitory concentration MIC (ug/mL) of *Baissea axillaries *leaves.

**Test organism**	**MIC (ug/mL) ^@ ^of aqueous extract**	**MIC (ug/mL) ^@ ^of ethanolic extract**
*Escherichia coli*	1.0 ± 0.16	0.8 ± 0.01
*Pseudomonas aeruginosa*	0.1 ± 0.00	0.8 ± 0.03
*Staphylococcus aureus*	ND	ND
*Streptococcus faecalis*	--	O.8 ± 0.05

@ Values are mean + SEM (n = 5).

ND Represents not done

Observation of the behaviours of the rats and general conditions showed loss of weight in rats administered *Baissea axillaries *compared with the control. This may be due to the fact that these rats experienced loss of appetite.

Although there was no incidence of mortality from the acute toxicity test, which was found to be above 5000 mg/kg, implying that the extract is relatively safe with low risk of acute intoxication, histological examinations of the liver of rats administered with 250 mg/kg showed mere ballooning degeneration. (The cytoplasm normally stains red but in ballooning degeneration, it stains white). At 500 mg/kg, the liver showed increased cytoplasmic eosinophilia and densely stained nuclei in some areas and fragmentation in others, compared to the control. These are evidence of liver cell damage. The testes at low and high doses showed ill-defined sertoli/leydig cells. There were indistinct cell outlines.

The tissues of the heart and kidney at both doses appeared normal. There were no evidences of tissue necrosis and no remarkable changes were observed.

This validated information should be useful to traditional healers and patients on the judicious use of *Baissea axillaries*, as its efficacy and safety can to an extent are guaranteed, having demonstrated a broad spectrum of antimicrobial activity and showed no remarkable toxicological effects at the dose of 250 mg/kg. However, there is the need for investigations into possible drug interactions of *Baissea axillaries *with antiretrovirals, to forestall possible treatment failure and viral resistance.

## Conclusion

Extracts of *Baissea axillaries *have demonstrated antimicrobial activity against clinical strains of selected microorganisms. While there is toxicity at the dose of 500 mg/kg, the therapy shows potential for application in the treatment of diarrhoea associated with AIDS/HIV. Further studies of *Baissea axillaries *on diarrhoea and toxicity are necessary to evaluate its mechanism of action and to fully establish its safety profile.

## Competing interests

The author(s) declare that they have no competing interests.

## Authors' contributions

TA carried out the Plant preparations, Antimicrobial and Phytochemical investigations while FA carried out the Toxicity evaluation.

**Table 3:** Results of preliminary Phytochemical Screening of *Baissea axillaries *leaves. Chromatographic results.

**Secondary Metabolites**		***Baissea axillaries extracts***			
		**Aqueous**		**Ethanolic**	
**Alkaloids**					
Dragendorff's reagent		+		+	
Wagner's reagent		+		+	
Mayer's reagent		+		+	
1% picric acid reagent		+		+	
**Anthracene derivatives**					
Combined anthraquinones		-		-	
Free anthraquinones		-		-	
**Tannins**					
Phenazone test		+		+	
Iron complex test		+		+	
Formaldehyde test		+		+	
Modified iron complex test		+		+	
**Saponin glycosides**					
Frothing test		-		-	
Haemolysis test		-		-	
**Cardiac glycosides**					
Keller killiani test		-		-	
Kedde test		-		-	
**Cyanogenetic glycosides**					
Sodium picrate test paper		+		+	

Thin layer chromatography					

**Extract**	**No of spots**	**Colour in daylight**	**Colour in UV**	**Colour after spraying with Dragendorff**	**R**_**f**_**value**

Aqueous	2	colourless	Light green fluorescent	reddish brown	0.55 0.72
Ethanolic	2	colourless	Light green fluorescent	reddish brown	0.53 0.71

Paper chromatography					

**Extract**	**No of spots**	**Colour in daylight**	**Colour in UV**	**Colour after spraying with Ferric chloride**	**R**_**f**_**value**

Aqueous	1	colourless	green fluorescent	Violet colour	0.73
Ethanolic	1	colourless	green fluorescent	Violet colour	0.72

Key:

-- (absent); + (present)

**Table 4:** Histological effects of the administration of ethanolic extracts of *Baissea axillaries *on Rats.

Dosage of administration	Histological effect on Liver tissues	Histological effect on Kidney tissues	Histological effect on Heart tissues	Histological effect on the testes
0.9 ml of normal saline	Tissues appeared normal. No evidence of tissue necrosis	Tissues appeared normal. No evidence of tissue necrosis	Tissues appeared normal. No evidence of tissue necrosis	Tissues appeared normal. No evidence of tissue necrosis
250 mg/kg of *Baissea axillaries*	Ballooning degeneration of the liver showing signs of early degenerative changes	Tissues appeared normal. No evidence of tissue necrosis	Tissues appeared normal. No evidence of tissue necrosis	Presence of ill defined testes with indistinct cell outlines
500 mg/kg of *Baissea axillaries*	Densely stained nuclei, increased congestion of the vessels all of which are evidence of tissue necrosis	Tissues appeared normal. No evidence of tissue necrosis	Tissues appeared normal. No evidence of tissue necrosis	Presence of ill defined testes with indistinct cell outlines

## Note

Acute toxicity test.

After intraperitoneal administration of extracts of *Baissea axillaries *in 20 mice up to 5000 mg/kg, no death was recorded.

## Pre-publication history

The pre-publication history for this paper can be accessed here:


